# Novel Strategies against Hepatocellular Carcinoma through Lipid Metabolism

**DOI:** 10.32604/or.2025.066440

**Published:** 2025-10-22

**Authors:** Yuanyuan Yang, Peipei Zhao, Hepu Chen, Yixuan Tu, Yujia Zhou, Xu Liu, Lyly Sreang, Zhigang Zhou, Jian Tu

**Affiliations:** 1 Guangxi Key Laboratory of Molecular Medicine in Liver Injury and Repair, The Affiliated Hospital of Guilin Medical University, Guilin, 541001, China; 2 College of Pharmacy, Guilin Medical University, Guilin, 541199, China; 3 Guangxi Key Laboratory of Diabetic Systems Medicine, Guilin Medical University, Guilin, 541199, China; 4 Department of Rheumatology and Immunology, The First Affiliated Hospital of Jinan University, Guangzhou, 510632, China; 5 Department of Anesthesiology, The Second Affiliated Hospital of Guilin Medical University, Guilin, 541199, China

**Keywords:** Hepatocellular carcinoma, lipid metabolism, fatty acid, sphingolipids, cholesterol

## Abstract

Hepatocellular carcinoma (HCC) is characterized by its highly invasive and metastatic potential, as well as a propensity for recurrence, contributing to treatment failure and increased mortality. Under physiological conditions, the liver maintains a balance in lipid biosynthesis, degradation, storage, and transport. HCC exhibits dysregulated lipid metabolism, driving tumor progression and therapeutic resistance. This review aims to elucidate the roles of fatty acid, sphingolipid, and cholesterol metabolism in HCC pathogenesis and explore emerging therapeutic strategies targeting these pathways. Key findings demonstrate that upregulated enzymes like fatty acid synthase (FASN), acetyl-CoA carboxylase (ACC), enhance *de novo* lipogenesis and β-oxidation, and promote HCC proliferation, invasion, and apoptosis evasion. Sphingolipids exert dual functions: ceramides suppress tumors, while sphingosine-1-phosphate (S1P) drives oncogenic signaling. Aberrant cholesterol metabolism, mediated by HMG-CoA reductase (HMGCR), liver X receptor α (LXRα), and sterol regulatory element-binding protein 1 (SREBP1), contributes to immunosuppression and drug resistance. Notably, inducing ferroptosis by disrupting lipid homeostasis represents a promising approach. Pharmacological inhibition of key nodes—such as FASN (Orlistat, TVB-3664), sphingomyelin synthase (D609), or cholesterol synthesis (statins, Genkwadaphnin)—synergizes with sorafenib/lenvatinib and overcomes resistance. We conclude that targeting lipid metabolic reprogramming, alone or combined with conventional therapies, offers significant potential for novel HCC treatment strategies. Future efforts should focus on overcoming metabolic plasticity and optimizing combinatorial regimens.

## 1 Introduction

Hepatocellular carcinoma (HCC), one of the most common solid malignant tumors, ranks sixth in incidence and third in mortality worldwide [1,2]. The liver is a central organ regulating energy metabolism, mainly depending on the balance of lipid metabolism [3]. Lipids not only serve as the primary energy storage substances, but also play crucial roles in cellular membranes [4]. Once this balance is disrupted, it could result in hepatic inflammation, fibrosis, and even cancer. The energy of lipid metabolism can promote the proliferation, invasion, and metastasis of HCC cells [5]. Additionally, bioactive metabolites and intermediates generated during lipid metabolism can modulate cellular signaling pathways and influence cytoskeletal organization. Recent studies have shown that targeting lipid metabolism is a promising approach for treating HCC [6,7]. The metabolic processes mediated by fatty acids, sphingolipids, and cholesterol are closely related to the pathogenesis of HCC [8].

HCC subtypes demonstrate marked heterogeneity in lipid metabolic functions across distinct molecular classifications and disease stages. These differences not only profoundly influence tumor proliferation, invasion, and metastasis but also are closely linked to therapeutic response and clinical prognosis. HCC cells predominantly rely on *de novo* lipogenesis (DNL) for energy metabolism, characterized by the synergistic activation of sterol regulatory element-binding protein 1 (SREBP1) and fatty acid synthase (FASN), which drives the synthesis of saturated fatty acids to fuel abnormal cancer cell proliferation. In contrast, intraepithelial lymphocytes (IELs) adopt an exogenous lipid uptake-dependent mode, where cluster of differentiation 36 (CD36)-mediated influx of long-chain fatty acids predominates. Concurrently, bile acid metabolic reprogramming enhances the conversion of primary bile acids to secondary bile acids, thereby promoting invasion and metastasis [9]. In addition, mixed liver cancer of those two types exhibits a unique “dual-driven” lipid metabolic phenotype, combining DNL and exogenous lipid uptake. This metabolic adaptability allows tumor cells to maintain proliferative advantages under microenvironmental nutrient fluctuations: DNL supplies raw materials for membrane phospholipid synthesis, while CD36-mediated lipid influx is transported via fatty acid binding protein 1(FABP1) to mitochondrial β-oxidation, sustaining ATP production and tumor growth [10].

In the early stages of HCC (I-II), tumor cells undergo lipid metabolic reprogramming, marked by significant activation of the DNL pathway [11], which drives the synthesis of nascent fatty acids to meet the demands of rapid proliferation [12]. During this phase, small, dispersed lipid droplets form in the cytoplasm, sequestering excess free fatty acids (FFA) and scavenging reactive oxygen species (ROS) to maintain redox homeostasis, thereby delaying the transition to an invasive phenotype. As the tumor progresses to stage III, enhanced mitochondrial β-oxidation activity dominates, fueling energy production and activating EMT-related pathways to promote cancer cell invasion and migration. At stage IV, pathological lipid droplets accumulate, which physically sequester lipophilic targeted agents like sorafenib, thereby reducing their effective intracellular concentration and contributing to drug resistance [13].

**Figure 1:**
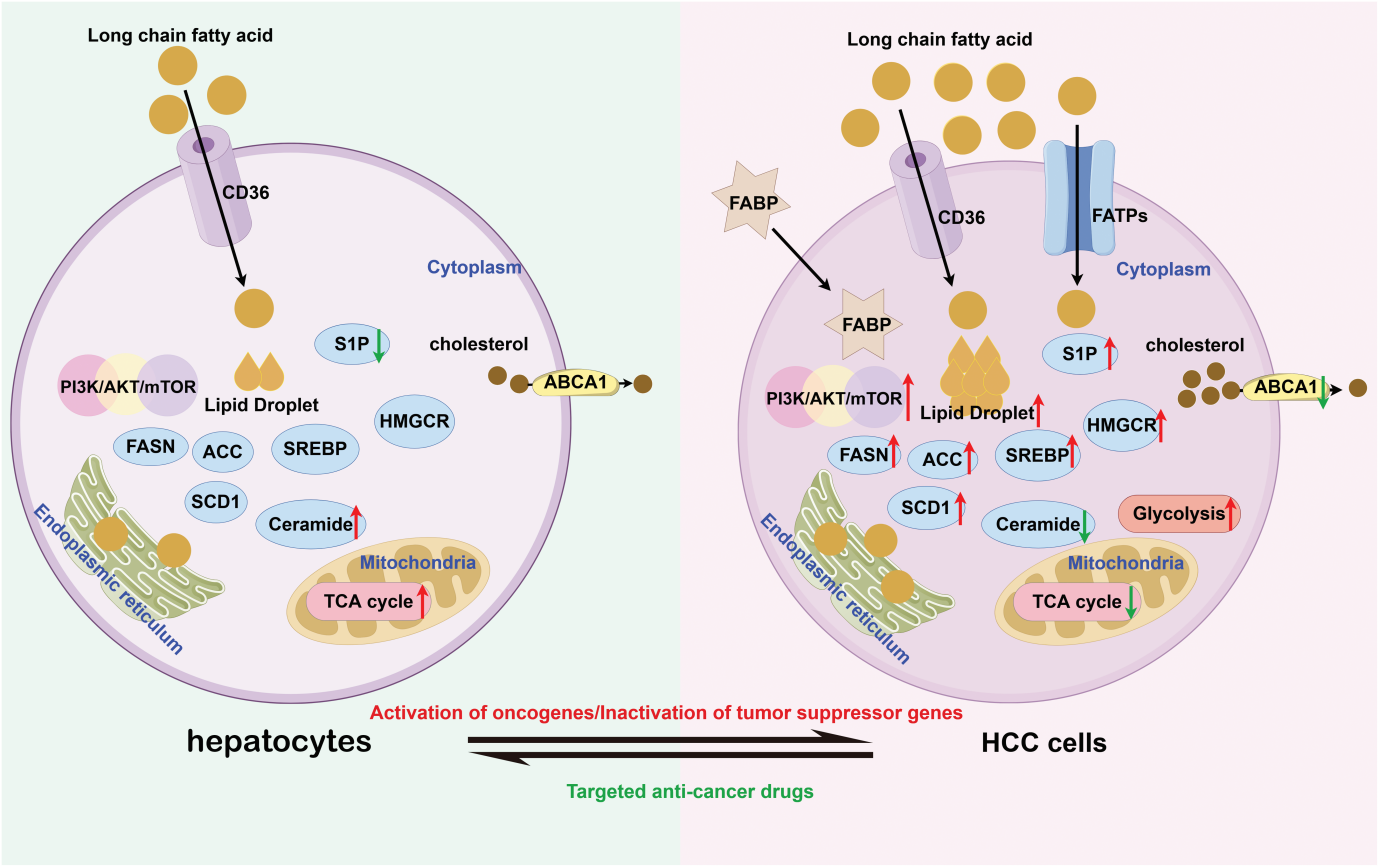
The difference of metabolism between hepatocytes and HCC cells (by Figdraw 2.0). Compared to hepatocytes, HCC cells not only enhance endogenous fatty acid synthesis capacity but also markedly increase exogenous fatty acid uptake to meet the metabolic demands of rapid proliferation. Notably, HCC cells exhibit aberrant upregulation in both expression and activity of lipid-synthesizing enzymes (e.g., FASN, ACC) and key signaling pathways, including PI3K/AKT/mTOR. In contrast, the activity of the ABCA1 transporter responsible for lipid efflux is markedly suppressed, leading to intracellular lipid accumulation and characteristic lipid droplet formation. From an energy metabolism perspective, while normal cells primarily rely on the TCA cycle for efficient ATP production, HCC cells demonstrate the Warburg effect by preferentially utilizing enhanced glycolytic pathways for energy supply. CD36, Cluster of Differentiation 36; S1P, Sphingosine-1-phosphate; FASN, Fatty Acid Synthase; ACC, Acetyl-CoA Carboxylase; SCD1, Stearoyl-CoA desaturase 1; SREBP, Sterol Regulatory Element-Binding Protein; HMGCR, HMG-CoA reductase; ABCA1, ATP-Binding Cassette Subfamily A Member 1; FABP, Fatty Acid-Binding Protein; FATPs, Fatty Acid Transport Proteins; TCA, Tricarboxylic Acid Cycle; PI3K, Phosphatidylinositol 3-Kinase; AKT, Protein Kinase B; mTOR, Mechanistic Target of Rapamycin; HCC, Hepatocellular carcinoma

The above suggests that targeting lipid metabolism might be an effective approach for the treatment of HCC. Compared with hepatocytes, HCC cells exhibit significant differences in lipid metabolism (Fig. 1). Therefore, this article reviews novel strategies against HCC through lipid metabolism, aiming to advance future diagnostic and preventive strategies for this malignancy.

## 2 Lipid Metabolism in HCC

Lipid metabolism is a complex process occurring at different levels in various organs and tissues. It requires the combined action of multiple genes and metabolic enzymes to achieve dynamic equilibrium. In the liver, lipids are mainly divided into eight categories: fatty acyl groups (including fatty acids), sphingolipids, sterols (including cholesterol), and so on [14,15]. Among them, fatty acid metabolism, sphingolipid metabolism, and cholesterol metabolism are closely related to HCC.

### 2.1 Fatty Acid Metabolism in HCC

Fatty acid metabolism can modulate the expression and activity of lipid metabolism enzymes as a result of the abnormal activation of oncogenic signaling pathways. This process may accelerate the occurrence and progression of HCC. Fatty acids are signal precursors that regulate metabolism during the development of HCC, and are also energy sources in cell proliferation, invasion/migration and apoptosis [16].

#### 2.1.1 Promoting Cell Proliferation

Fatty acids can promote cell cycle progression, thereby increasing the proliferation rate of cells. They could activate certain signaling pathways, such as the phosphatidylinositol 3-kinase pathway (PI3K/Akt), which promotes cell survival and proliferation. Furthermore, the most typical feature of HCC is the upregulation of FA synthesis-related genes, and high expression of FASN usually indicates poor prognosis. The study found that knocking out FASN significantly inhibited HCC driven by Akt activation in a mouse model [17]. The study also confirmed that linear free energy (LFE) could upregulate the relative expression levels of genes related to the PI3K/Akt pathway and fatty acid metabolism [18]. Peroxisome proliferator-activated receptor c (PPARc) belongs to the peroxisome proliferator-activated receptor gamma coactivator 1 (PGC-1) coactivator family and is considered a major regulator of mitochondrial biosynthesis, oxidative metabolism, and antioxidant defense [19]. The coactivators PGC-1α and PGC-1β display comparable expression patterns and are significantly expressed in tissues characterized by heightened mitochondrial energy metabolism [20]. Additionally, peroxisome proliferator-activated receptor α (PPARα) governs the constitutive transcription of genes that encode enzymes involved in fatty acid transport. Cytochrome P450 consists of a group of ω-hydroxylase enzymes that convert fatty acids into forms suitable for mitochondrial uptake, facilitating their transport to mitochondria for energy production. This process not only eliminates excess FFA but also contributes to the synthesis of bioactive fatty acid molecules [21]. The decrease in cytochrome p450 family 4 (CYP4) expression is associated with liver fat accumulation [22]. CYP4A, CYP4B, and CYP4F, together with CYP4V, metabolize short-chain fatty acids, medium-chain fatty acids, and long-chain fatty acids, respectively. Among them, CYP4F2, CYP4F12, and CYP4V2 are significantly positively correlated with lipid metabolism pathways, and their functional components contribute to HCC progression through diverse metabolic mechanisms [23]. A study that analyzed gene expression profiles in the liver and serum of HCC patients suggests that the lncRNA RP11-466I1 is involved. It may increase FA uptake and promote the occurrence of HCC by upregulating PPAR γ and FA metabolism-related gene LPL [24]. A study found that the inactivation of fatty acid synthase could downregulate the expression level of 5-lipoxygenase (5-LOX) in HepG2 cells and reduce the content of leukotriene B4 (LTB4) in culture medium and cell lysates. This indicates that hepatitis B virus X protein with deletion at residue 127 (HBx Δ 127) promotes cell growth in liver cancer cells through a positive feedback loop involving fatty acid synthase (FAS) and 5-LOX [25].

#### 2.1.2 Increasing Cell Invasion/Migration

HCC metastasis represents a clinically critical stage associated with dismal patient prognosis [26,27]. Given the tight pathophysiological interplay between fatty acid metabolism and hepatic function, identifying biomarkers and therapeutic targets in this context is imperative [28]. Saturated fatty acids, for instance, can promote cancer cell invasion, possibly mediated by altering membrane fluidity and permeability. The protein arginine methyltransferase 1-9 (PRMT1-9) governs protein arginine methylation, an essential post-translational modification pathway that dynamically regulates cellular signaling. By suppressing cell viability, migration, and invasion, PRMT1 knockout in HCC cells concurrently reduces expression of genes involved in fatty acid metabolism. Furthermore, PRMT1-coexpressed genes are enriched in fatty liver diseases and drug-induced liver injury, with functional links to fatty acid metabolism [29–31]. PRMT1 accelerates hepatocellular carcinogenesis through immune microenvironmental reprogramming and fatty acid metabolic dysregulation. Acyl-coenzyme A thioesterase 9 (ACOT9), a pivotal gatekeeper of intracellular fatty acid flux, cleaves acyl-CoA thioesters to liberate free fatty acids and coenzyme A. ACOT9 drives hepatocellular carcinoma progression by reprogramming lipid metabolism, emerging as a promising therapeutic target in HCC [32]. As a secreted acid-phosphorylated glycoprotein, Tuftelin 1 (TUFT1) is pathologically overexpressed during hepatocarcinogenesis and highly correlated with poor patient survival and aggressive tumor phenotypes [33]. TUFT1 modulates fatty acid metabolism to drive intracellular lipid deposition in HCC cells, while demonstrating physical interaction with the lipid metabolic regulator CREB1. TUFT1 could also regulate the activity of CREB1 and the transcription of key enzymes involved in lipid production. TUFT1 significantly promotes HCC cell proliferation, partially reversed by treatment with CREB1 inhibitor KG-501. In addition, TUFT1 promotes the ability of HCC cells to invade *in vitro*. Research has shown that CD147 overexpression triggers AKT-mTOR cascade activation, potentiating SREBP1c transcriptional output [34]. SREBP1c transactivation elevates FASN/ACC expression, propelling hepatocellular carcinoma progression and metastasis. In addition, the reduction of krüppel-like factor 5 (KLF5) levels showed the reverse of epithelial-mesenchymal transition (EMT) via PI3K/AKT signaling and the decreased expression of MMP2/ MMP9 in HCC cells both *in vitro* and *in vivo* [35]. In addition, FA could also influence the progression of HCC by regulating signal prerequisites and serving as an energy source.

Research has identified miR-377-3p as a key regulator of carnitine palmitoyl transferase 1C (CPT1C) expression and lipid metabolism [36]. Through 3’-UTR targeting-mediated CPT1C downregulation, miR-377-3p attenuates fatty acid β-oxidation, thereby curbing HCC oncogenicity (proliferation, migration, invasion, metastasis) across *in vitro* and *in vivo* systems. Additionally, pyruvate dehydrogenase kinase 4 (PDK4) knockdown triggers *de novo* lipogenesis by upregulating rate-limiting enzymes FASN and SCD in HCC cells, which could inhibit cell migration [37]. An experiment has confirmed that the silence of solute carrier family 25 member 19 (SLC25A19) and FASN potently curbs oncogenic proliferation and migratory capacity [38]. Recent research found that a DNA methyltransferase 1 (DNMT1) inhibitor effectively increases acyl-CoA synthetase medium-chain family member 5 (ACSM5) expression and reduces promoter region methylation [39]. ACSM5 overexpression in Huh7 cells attenuated fatty acid accrual and malignant phenotypes (proliferation/migration/invasion) *in vitro*, while suppressing xenograft tumorigenesis *in vivo*. Furthermore, ACSM5 overexpression also decreased signal transducer and activator of transcription 3 (STAT3) phosphorylation, subsequently affecting downstream cytokine transforming growth factor-β (TGFB) and fibroblast growth factor 12 (FGF12) messenger ribonucleic acid (mRNA) levels.

All cellular activities require the provision of energy. Fatty acids are one of the major sources of energy for cells. In rapidly proliferating cells, such as cancer cells, the demand for energy is particularly high. Therefore, the supply of fatty acids is crucial for supporting the growth and survival of cancer cells. Research has shown that the methyltransferase-like 5 (METTL5)- acyl-CoA synthetase long chain family member 4 (ACSL4) axis promotes β-oxidation [40]. The ACSL family plays an important role in fatty acid metabolism in cancer [41,42]. The ACSL family of proteins has a dual function of promoting *de novo* adipogenesis and β-oxidation, thereby promoting cancer growth and progression [43,44]. Beyond β-oxidation facilitation, ACSL isoforms orchestrate lipogenesis and lipid droplet biogenesis via transcriptional reprogramming. ACSL4 potentiates METTL5-driven fatty acid metabolism and HCC progression, while dual targeting synergistically suppresses hepatocarcinogenesis *in vivo*. METTL5- tRNA methyltransferase 112 (TRMT112)-mediated 18S rRNA N^6^-methyl adenosine (m6A) modification promotes HCC growth and metastasis *in vitro* and *in vivo*. Mechanistically, 18S rRNA m6A modification promotes the assembly and translation of 80S ribosomes involved in HCC fatty acid metabolism [45,46]. In addition, targeting METTL5 and fatty acid metabolism may synergistically inhibit the occurrence of HCC tumors *in vivo*. METTL5 promotes *de novo* fat generation and fatty acid beta oxidation processes. An increasing number of studies indicate that *de novo* adipogenesis and fatty acid oxidation are simultaneously activated and coordinated to promote cancer progression [47,48].

#### 2.1.3 Resisting Cell Apoptosis

**Figure 2:**
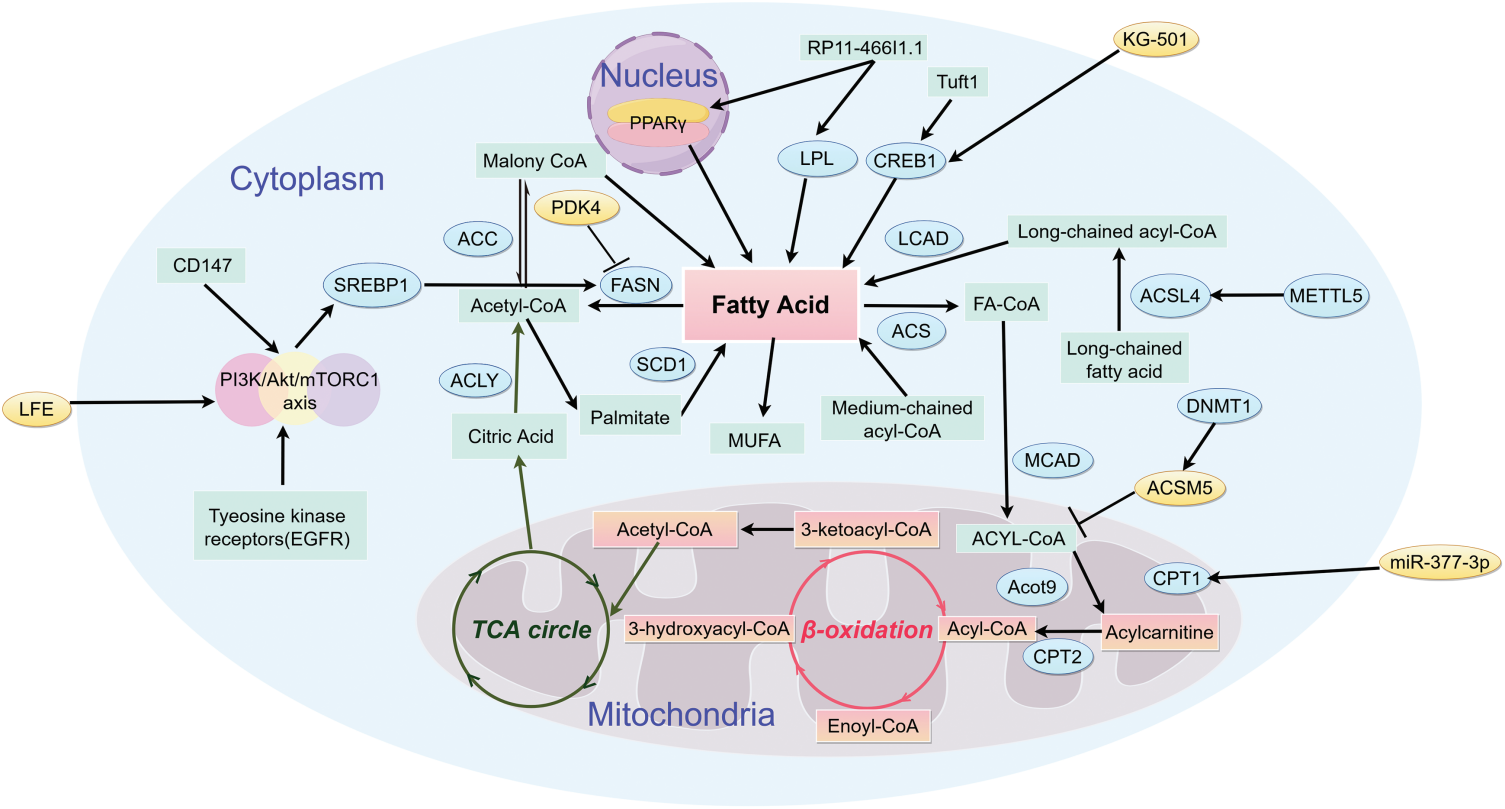
Metabolic pathways of fatty acid in HCC (by Figdraw 2.0). Fatty acid catabolism critically regulates metabolic reprogramming throughout hepatocarcinogenesis. The initial flux-controlling step, mediated by mitochondrial outer membrane-bound CPT1, catalyzes acyl-CoA conversion to acylcarnitine—essential for mitochondrial acyl-CoA import. In contrast, medium/short-chain acyl-CoAs diffuse freely across the inner membrane. Subsequent carnitine/acylcarnitine translocase-facilitated transport delivers acylcarnitine to the matrix, where CPT2 regenerates acyl-CoA. Cytosolic ACLY converts TCA cycle-derived citrate to acetyl-CoA, which ACC1 carboxylates into malonyl-CoA—the cytoplasmic rate-limiting precursor. FAS then condenses 7 malonyl-CoA molecules yielding palmitate, fueling ATP/NADH production via mitochondrial β-oxidation and TCA cycling. CD147, Cluster of Differentiation 147; SREBP1, sterol regulatory element-binding protein 1; ACC, Acetyl-CoA Carboxylase; ACLY, ATP Citrate Lyase; FASN, Fatty Acid Synthase; SCD1, Stearoyl-CoA Desaturase 1; LPL, Lipoprotein Lipase; CREB1, cAMP Responsive Element Binding Protein 1; LACD, Long-chain acyl-CoA dehydrogenase; ACS, Acyl-CoA Synthetase; MCAD, Medium Chain Acyl-CoA Dehydrogenase; Acot9, Acyl-CoA Thioesterase 9; CPT2, Carnitine Palmitoyl Transferase 2; CPT1, Carnitine Palmitoyl Transferase 1; DNMT1, DNA Methyltransferase 1; ACSL4, Acyl-CoA Synthetase Long-Chain Family Member 4; METTL5, Methyltransferase Like 5

Fatty acids may inhibit cell apoptosis, thereby prolonging the lifespan of cancer cells. Research suggests that the absence of FASN only delays the occurrence of tumors, indicating the existence of other mechanisms that promote HCC cell proliferation and survival [49]. Recent research demonstrates that an increase in monounsaturated fatty acids contributes to the *de novo* synthesis of fatty acids in liver cancer cells [50]. The role of SCD1 in HCC is related to the regulation of p53 protein (P53), WNT/β-catenin, epidermal growth factor receptor (EGFR), and autophagy [51]. CD147 upregulation triggers AKT-mTOR signaling, thereby increasing sterol regulatory element-binding SREBP1c expression [52]. The upregulation of SREBPlc levels increases the expression of FASN and ACC, leading to tumor growth and metastasis. The upregulation of CD147 also reduces PPAR α, and downregulates CPT1A and ACOX1, leading to HCC growth and metastasis [53]. High expression of thyroid hormone receptor interactor protein 13 (TRIP13) in liver cancer affects survival rate and is associated with enrichment of certain molecules in the processes of RNA degradation and fatty acid metabolism. The increased expression of TRIP13 in liver cancer tissues is associated with liver cancer progression. Silencing TRIP13 may inhibit cell viability, migration, and invasion, and induce cell apoptosis. Knocking down TRIP13 could also inhibit tumor formation *in vivo* [54]. In HCC, the FA metabolic pathway involves the mitochondrial breakdown of long-chain fatty acids, which are oxidized to generate acetyl-CoA and subsequently fuel the tricarboxylic acid cycle (TCA), a critical step in cellular energy production (Fig. 2).

### 2.2 Sphingolipid Metabolism in HCC

**Figure 3:**
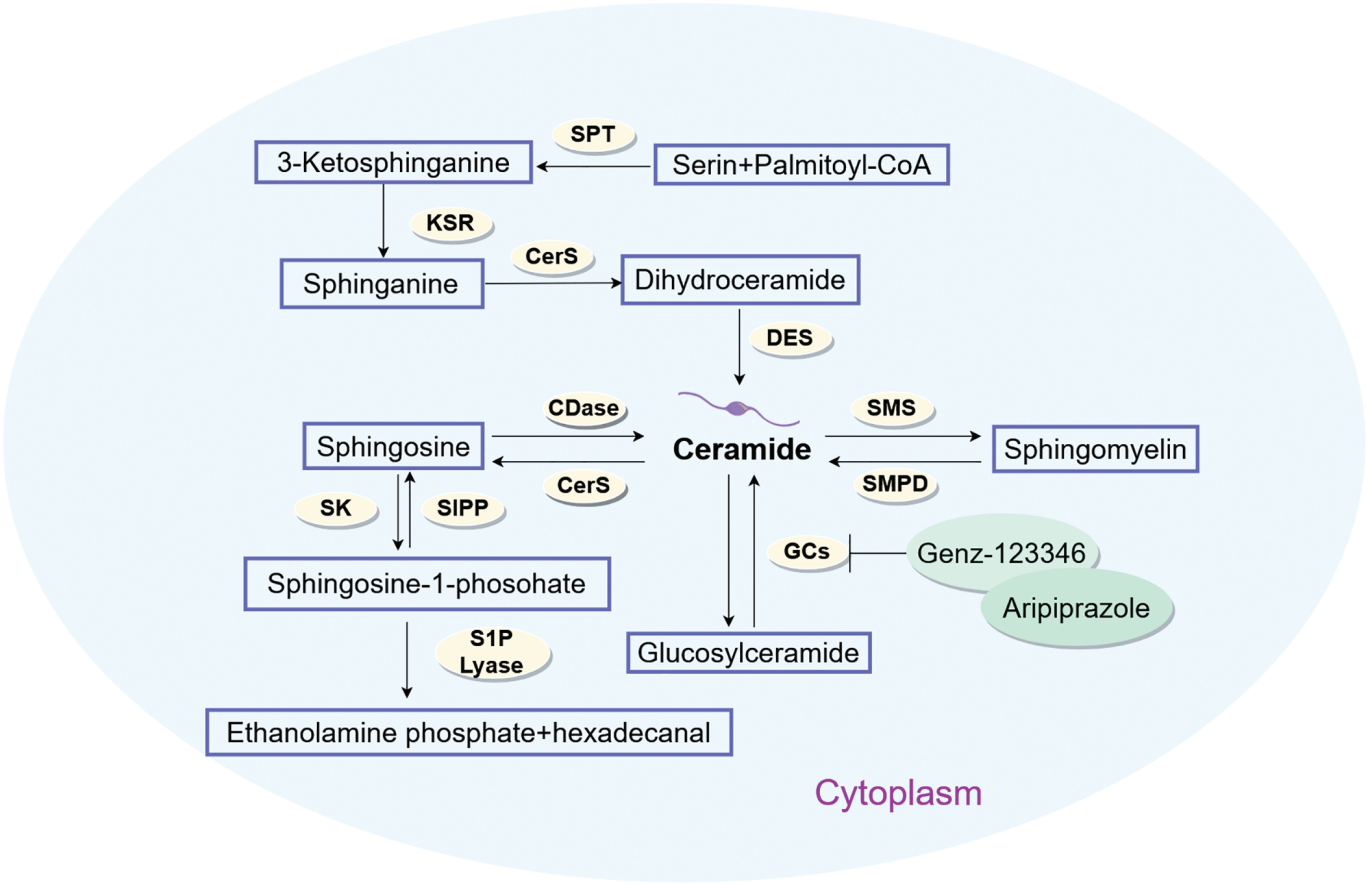
Metabolic pathways of sphingolipid in HCC (by Figdraw 2.0). Ceramides serve as pivotal metabolic integrators in sphingolipid biotransformation, biosynthesized through *de novo* pathways via SPT-CerS-DES enzymatic cascades, sphingomyelinase-mediated membrane sphingomyelin hydrolysis, or glucosylceramidase-catalyzed complex sphingolipid catabolism. SPT, Serine Palmitoyl Transferase; KSR, 3-Ketosphinganine Reductase; CerS, Ceramide Synthase; DES, Dihydroceramide Desaturase; CDase, Ceramidase; SMS, Sphingomyelin Synthase; SMPD, Sphingomyelin Phosphodiesterase; GCs, Gangliosides; S1PP, Sphingosine-1-Phosphate Phosphatase; SK, Sphingosine Kinase; S1P Lyase, Sphingosine-1-Phosphate Lyase

Bioactive sphingolipids—including ceramides, sphingosine, C1P, and S1P—critically modulate hepatocellular carcinoma cell fate decisions, such as proliferation, senescence and apoptosis [55,56]. As the metabolic nexus of sphingolipids, ceramides orchestrate their biotransformation. Endogenous ceramide biosynthesis proceeds via the *de novo* route through serine palmitoyl transferase (SPT), ceramide synthase (CerS), and dihydroceramide desaturase (DES) [57] sphingomyelinases (SMase) and glucosylceramidase-mediated enzymatic hydrolysis liberates ceramides from membrane sphingomyelins (SMs) or complex sphingolipids [58]. CerS reacylates sphingosine—a sphingolipid catabolic intermediate—recycling it into ceramides. Ceramides may accumulate briefly or serve as precursors for sphingolipids like C1P, S1P, and glucosylceramide (GlcCer). They can also be recycled back into sphingomyelins (SMs) [59]. The enzymatic conversion of phosphorylcholine and ceramide into sphingomyelins (SMs) is mediated by sphingomyelin synthetase [60]. Glycosphingolipid biosynthesis from ceramides occurs in the Golgi apparatus (GA), where specific glycosyltransferases mediate glycosylation [61]. Additionally, glycosylation of sphingolipids occurs within lysosomes. Concurrently, ceramide kinase phosphorylates ceramide in the GA. As central metabolites in sphingolipid pathways, ceramides exert anti-proliferative effects by suppressing tumor cell proliferation/migration while inducing autophagy and apoptosis. Conversely, sphingosine-1-phosphate (S1P) and related sphingolipids demonstrate oncogenic properties, driving malignant progression through tumor cell transformation, motility, proliferation, and chemoresistance induction. Research indicates that in ceramide-refractory malignancies, sphingolipid metabolic reprogramming diverts exogenous ceramides toward pro-survival sphingolipid synthesis, enabling acquired ceramide resistance [62,63]. Clinical evidence indicates upregulated sphingolipid expression in HCC tissues, suggesting metabolic dysregulation linked to hepatocarcinogenesis [64]. Within this pathway, the SPHK1/S1P signaling axis functions as a pivotal oncogenic driver, with SPHK1 overexpression established as a consistent biomarker in hepatic malignancies [65]. SHPK1 knockdown perturbs sphingolipid homeostasis, characterized by depleted sphingosine 1-phosphate (S1P), accumulated ceramides, and suppressed cellular viability [66]. A study has shown that Genz-123346 and aripiprazole synergistically suppress Huh7/Hepa1-6 HCC cell proliferation and tumor microsphere expansion [67] (Fig. 3).

### 2.3 Cholesterol Metabolism in HCC

Emerging evidence implicates cholesterol transport in hepatocellular carcinoma (HCC) pathogenesis, driving malignant progression through proliferation, metastasis, and chemoresistance [68]. Notably, dysregulated cholesterol metabolism in HCC exhibits aberrant synthetic pathways that constitute critical oncogenic mechanisms for tumor growth [69].

**Figure 4:**
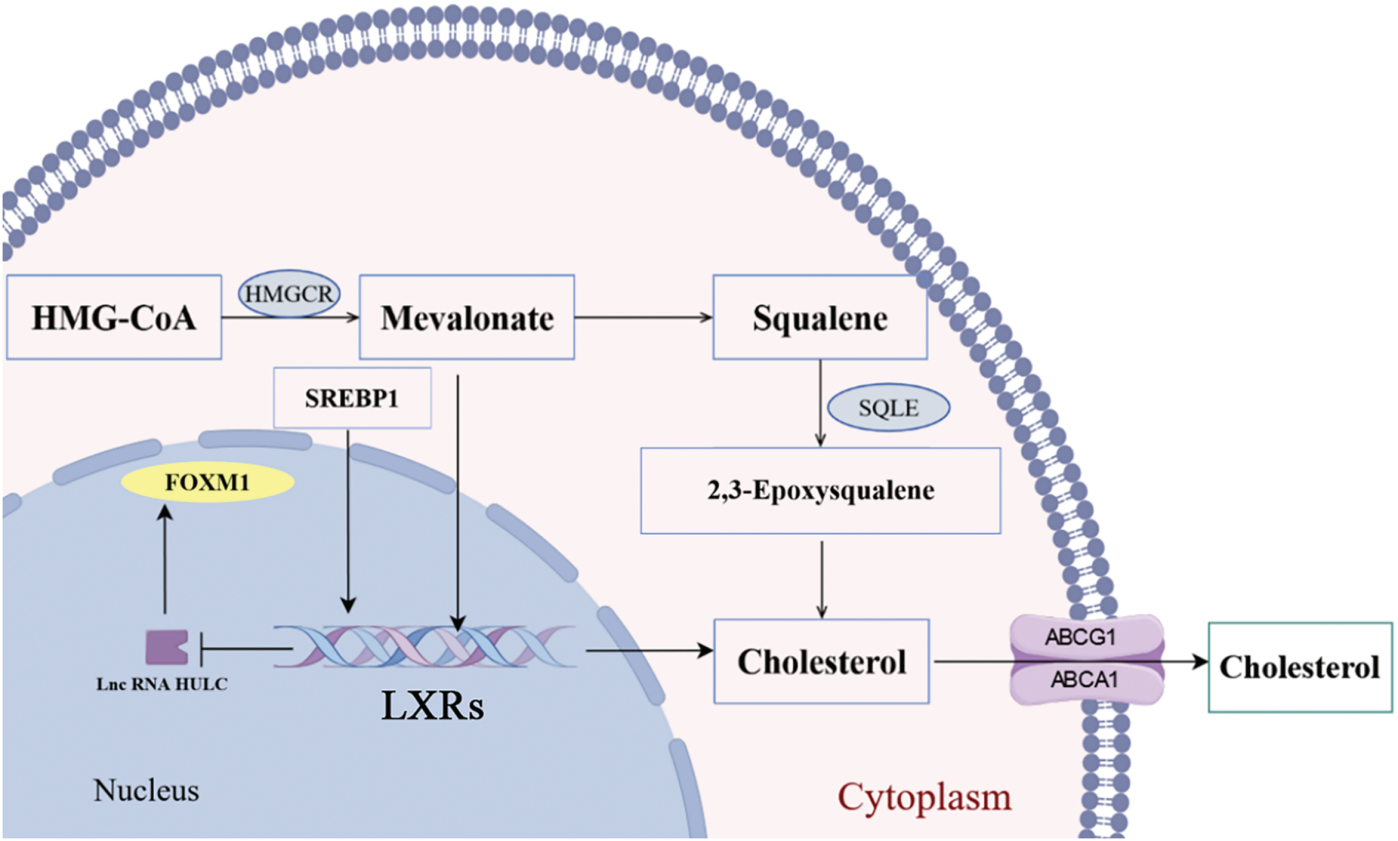
Metabolic pathways of cholesterol in HCC (by Figdraw 2.0). HMGCR is a rate-limiting enzyme for cholesterol synthesis in the mevalonate pathway, affecting the synthesis of mevalonate and acting on LXRs, which could be blocked by statins. Furthermore, LXRs functions to regulate cholesterol homeostasis by transactivation of metabolic players as SREBP1 and FASN. HMGCR, 3-Hydroxy-3-Methylglutaryl-CoA Reductase; SQLE, Squalene Epoxidase; SREBP1, sterol regulatory element-binding protein 1; FOXM1, Forkhead Box Protein M1; LXRs, Liver X Receptors; ABCG1, ATP-Binding Cassette Sub-family G Member 1; ABCA1 ATP-Binding Cassette Sub-family A Member 1

Liver X receptor α (LXRα) mediates transcriptional activation upon binding cholesterol and its oxidized derivatives, promoting cholesterol conversion to bile acids [70]. Current evidence indicates that synthetic LXRs agonists demonstrate anti-proliferative effects and modulate progression phenotypes—including growth, invasion, and metastasis in malignant tumors [71,72]. Collectively, these findings establish LXRα as a pivotal regulatory hub in oncogenesis. Mechanistically, LXRα constrains TGF-β signaling activation to suppress HCC proliferation. Contemporary studies further confirm its potent anti-neoplastic effects, significantly impairing metastatic competence through reduced invasion and migration capacities [73]. Our research group has also conducted studies on LXRs in HCC. We found that LXR alpha and high expression of liver cancer transcript (highly upregulated in liver cancer, HULC) cut the HULC promoter region, combining expression, thereby lowering fork frame M1 (FOXM1) expression [74]. However, FOXM1 could activate c-myc promoter and promote the proliferation of liver cancer cells [75]. Notably, HMGCR silencing downregulates FOXM1 expression, implicating cholesterol biosynthesis in transcriptional control during HCC. Bergamottin (a natural LXRα agonist) upregulates ABCA1 transporter activity, enhancing cholesterol efflux and reducing intracellular lipid droplet accumulation in hepatoma cells [76]. Numerous preclinical studies have demonstrated that liver cholesterol has a tumorigenic effect in promoting the transition from non-alcoholic steatohepatitis (NASH) to HCC [77,78]. Cholesterol critically modulates membrane fluidity—thereby regulating protein functionality—through its role as a key membrane rheostat [79]. Metabolic syndrome-induced cholesterol accumulation disrupts plasma and organelle membrane integrity. Mitochondrial cholesterol enrichment reduces membrane fluidity, impairing electron transport chain function. This triggers ROS overproduction, lipid peroxidation, hepatocyte necrosis, and apoptosis—collectively constituting established HCC risk factors [80]. Cholesterol critically regulates invariant natural killer T (iNKT) cell activation—effectors with intrinsic anti-tumor capacity. Membrane cholesterol levels further modulate CD8^+^ T cell activity and PD-1/PD-L1 axis suppression. In the tumor microenvironment, excessive cholesterol consumes CD8^+^ T cells by regulating the expression of X-box binding protein 1 (XBP1), which in turn activates a series of endoplasmic reticulum stress related pathways that impair the function and induce apoptosis of CD8^+^ T cells and promotes the immune escape of tumor cells [81]. DDX39B drives hepatocellular carcinoma progression by activating SREBP1-dependent *de novo* lipogenesis, establishing its dual utility as a prognostic biomarker and therapeutic target [82]. Overall, the cholesterol load on the membrane system could increase the risk of HCC through multi-level mechanisms (Fig. 4).

### 2.4 Other Lipid Metabolism in HCC

Glycerophospholipid metabolism also plays an important role in HCC [83]. Studies have shown that in the early stages of HCC development, glycerophospholipid metabolism may have been disrupted, and phospholipid levels are positively correlated with tumor burden [84,85]. Some studies have found that both lysophosphatidyl choline and lysophosphatidyl ethanolamine continue to increase in liver cancer [86,87]. The reason for this phenomenon may be that lysophosphatidylcholine (LPC) is a structural unit of cell membrane glycerophospholipids, and the increase of these metabolites may reflect the high metabolic demand of HCC, which is consistent with the increased demand for glycerophospholipids in liver cancer.

Fat-soluble vitamins, including vitamin D, could regulate the process of HCC [88–90]. *In vitro* experiments have shown that vitamin D plays an anti-tumor role in HCC and could regulate the growth/progression of HCC by regulating the cell cycle and inhibiting mTOR. *In vivo*, vitamin D could regulate the progression of HCC, thereby activating cell apoptosis, reducing oxidative stress, and inhibiting inflammation [91–93].

## 3 New Strategies of HCC Treatment

With the increasing incidence rate and mortality of HCC, it is urgent to explore new treatment strategies. Numerous studies have shown that changes in lipids significantly affect the efficacy of drugs [94,95]. Hcc cells exhibit profound metabolic reprogramming distinct from normal hepatocytes, wherein dysregulated lipid metabolism fuels bioenergetic demands for proliferation and metastasis while generating onco-signaling mediators that perturb cellular architecture; consequently, targeting lipid metabolic vulnerabilities represents a promising HCC therapeutic strategy.

Clinically, however, current clinical trials in HCC face multiple challenges. Drug resistance remains a major hurdle, exemplified by platelet-derived growth factor receptor alpha (PDGFRA)/c-Jun pathway activation or lipid metabolism reprogramming leading to targeted therapy failure, while immunotherapy efficacy is limited by tumor microenvironment suppression such as CD8^+^ T-cell exhaustion [96]. Patient stratification lacks standardization; although multi-omics studies like proteogenomics have proposed subtypes such as metabolism-driven and microenvironment-dysregulated HCC, clinical translation is hindered by tumor heterogeneity and the absence of reliable biomarkers, including the limited prognostic utility of PD-L1 expression in HCC [97,98]. Combination strategies such as hepatic arterial infusion chemotherapy (HAIC) combined with targeted immunotherapy or stereotactic body radiotherapy (SBRT) with sorafenib demonstrate high conversion rates or survival benefits in single-arm trials but lack head-to-head comparisons and large-scale randomized validation [99]. Additionally, novel therapies like T cell receptor-engineered T cells (TCR-T) cell therapy or ultrasound-activated artificial enzyme-gene combinations show promise but require resolution of technical limitations and long-term safety assessments [100].

### 3.1 New Strategies for Treating HCC from the Perspective of Ferroptosis Pathways for HCC

Ferroptosis refers to iron-dependent, and regulatory necrosis mediated by lipid peroxidation, which is closely related to the occurrence and development of various cancers. Research has shown that the ferroptosis process of HCC cells is regulated by multiple signaling pathways and cytokines [101]. Inducing ferroptosis is of great significance in the treatment of HCC. Ferroptosis could regulate the growth of malignant tumors and has shown significant advantages in the treatment of malignant tumors. In HCC, changes in lipid metabolism are crucial for regulating ferroptosis [102]. Compared with normal cells, cancer cells have higher levels of iron demand and lipid metabolism, and lipid metabolism is widely present during ferroptosis [103]. Next, we will discuss how lipid metabolism affects the process of ferroptosis from three pathways: fatty acid metabolism, sphingolipid metabolism, and cholesterol metabolism.

#### 3.1.1 Ferroptosis of Fatty Acid Metabolism

Ferroptosis is a novel form of programmed cell death that has garnered significant attention in cancer treatment research in recent years [104]. Cellular fatty acid uptake is orchestrated by specialized transporters—including fatty acid translocase (FAT/CD36), fatty acid transport proteins (FATPs), and fatty acid-binding proteins (FABPs). CD36-mediated fatty acid internalization correlates with metastatic progression, where elevated transporter expression enhances oncogenic dissemination. Notably, CD36-overexpressing neoplastic cells preferentially store internalized fatty acids over oxidative utilization, potentially inducing ferroptotic vulnerability [105]. On the other hand, CD36 could inhibit ferroptosis by outputting trihydroxy arachidonic acid (AA) [106]. Beyond CD36, fatty acid transport protein 2 (FATP2) functionally complements fatty acid internalization. Pharmacological FATP2 inhibition delays oncogenic progression, whereas genetic ablation impairs arachidonic acid (AA) uptake, rendering cells ferroptosis-resistant [107]. Ferroptosis progression is pathognomonically driven by lipid peroxide accrual. Convergent contributions from iron dyshomeostasis, polyunsaturated fatty acid (PUFA) biogenesis, and peroxidation chain reactions propagate PUFA-peroxide generation. Critically, arachidonic acid (AA) and adrenic acid (AdA) serve as essential precursors for PUFA synthesis. Monounsaturated fatty acids (MUFAs) and associated lipid droplets confer ferroptosis resistance by competitively suppressing PUFA biosynthesis [108]. Nicotinamide adenine dinucleotide phosphate (NADPH) has been shown to prevent lipid damage and combat ferroptosis [109]. The cystine/glutamate antiporter/glutathione/glutathione peroxidase 4 (Xc/GSH/GPX4) axis and ferroptosis suppressor protein 1/dihydroorotate dehydrogenase/coenzyme Q_10_ (FSP1/DHODH/CoQ10) axis of the system could neutralize peroxides via free radical trapping [110].

#### 3.1.2 Ferroptosis of Sphingolipid Metabolism

A study had shown that glutamate-induced decrease in intracellular GSH could lead to activation of acid sphingophospholipase and upregulation of sphingosine levels, thereby inhibiting mitochondrial respiratory chain, promoting ROS generation, opening of mitochondrial permeability transition pores, and ferroptosis [111]. As a pleiotropic gene, sirtuin 3 (SIRT3) could regulate various cell death pathways through stimulation and specific substrate targeting, such as the acid sphingophospholipase/sphingosine-mediated ferroptosis pathway, thereby exerting a protective effect. Another research has found the relationship between sphingolipids and ferroptosis, and acid sphingophospholipase mediated redox activation activated autophagic degradation of GPX4, ultimately leading to lipid peroxidation and ferroptosis [112].

#### 3.1.3 Ferroptosis of Cholesterol Metabolism

A recent study [113] found that dysregulation of cholesterol homeostasis could lead to resistance to ferroptosis, thereby increasing the tumorigenicity and metastasis of cancer. Previous studies have shown that the precursor of cholesterol, 7-dehydrocholesterol (7-DHC), has a higher redox activity and could resist ferroptosis by directly inhibiting lipid peroxidation [114,115]. B7 homolog 3 (B7H3) ablation disrupts cholesterol homeostasis through AKT/SREBP2 hyperactivation, depleting membrane polyunsaturated phospholipids and sensitizing HCC cells to ferroptosis [116]. Researchers found that high cholesterol could lead to the resistance of cancer cells to ferroptosis and increase their tumorigenicity and metastasis. During metastatic dissemination, cholesterol accumulation in migratory HCC cells suppresses phospholipid peroxidation by stabilizing membrane PUFAs, thereby conferring ferroptosis resistance absent in static populations. However, in the late stage of liver cancer, when extensive liver damage occurs, higher cholesterol may indicate better preservation of liver function. In this case, the study may conclude that the higher cholesterol could inhibit the occurrence and development of liver cancer. In addition, in diagnosed liver cancer, an increase in intracellular cholesterol may have harmful effects on a cell type (such as tumor cells), but may promote the immune surveillance function of immune cells, thereby exhibits an overall beneficial effect [117].

### 3.2 New Strategies for Treating HCC from the Perspective of Fatty Acid Metabolism

Research found that the lipid metabolism of HCC cells was downregulated by the ketogenic rate-limiting enzyme 3-hydroxymethylglutaryl CoA (HMGCS2), thereby increasing the synthesis of fatty acids [118]. HMGCS2-knockdown tumors exhibited accelerated growth under ketogenic diet (KD) conditions, concomitant with elevated lipogenic markers and tumor weight-lipid content correlation. This demonstrates that HMGCS2 suppression enhances hepatic *de novo* lipogenesis via ketogenesis perturbation, compromising KD-mediated oncosuppression. Mechanistically, HMGCS2 governs HCC proliferation/migration through apoptosis modulation, c-Myc/cyclin D1 axis, and EMT pathway regulation—operating in a β-hydroxybutyrate-dependent manner [119]. In HMGCS2-expressing HCC, KD upregulates HMGCS2 expression, amplifying ketogenesis to constrain tumor proliferation. Orlistat, as a FASN inhibitor, could regulate fat metabolism by inhibiting FA synthesis, reduce HCC resistance to sorafenib, and improve drug efficacy [120]. Fatty acid transporter-5 (FATP5/SLC27A5) orchestrates fatty acid trafficking while constraining HCC invasive-metastatic cascades and epithelial-mesenchymal transition (EMT). Notably, synergistic targeting of nuclear factor erythroid 2-related factor 2 (NRF2) and thioredoxin reductase 1 (TXNRD1) with sorafenib—using bromosulfophthalein and auranofin—potentiates therapeutic vulnerability in FATP5-deficient malignancies [121,122]. After using the fatty acid β-oxidation (FAO) inhibitor etomoxir, the resistance of HCC to sorafenib significantly improved. Another FASN inhibitor, TVB3664, has limited efficacy as a single drug, but it significantly improves the efficacy of cabozantinib and sorafenib in the treatment of HCC [123].

### 3.3 New Strategy for Treating HCC from the Perspective of Sphingolipid Metabolism

Ultrasmall lipid nanoparticles (UsLNPs) engineered with phospholipid matrices and tumor-targeting peptides enable precision sorafenib delivery to murine neoplastic cells, eliciting potent therapeutic efficacy [124]. Sphingophospholipid metabolism critically governs HCC pathogenesis and chemoresistance. Sorafenib treatment potently upregulates sphingomyelin synthase 1 (SMS1) in HCC models, attenuating drug cytotoxicity. Consequently, SMS1 inhibitor D609 synergistically enhances sorafenib efficacy by suppressing rat sarcoma viral oncogene homolog (RAS) signaling [125]. S1P generation by SK2 promotes oncogenic survival. Synergy between sorafenib and SK2 inhibitor ABC294640 improves anti-tumor activity, whereas bavituximab targeting phosphatidylserine exerts dual anti-angiogenic and immunostimulatory effects [126]. Emerging evidence correlates acquired sorafenib resistance with profound phosphatidylcholine remodeling in tumor tissues. This suggests that phosphatidylcholine has the potential as a biomarker for sorafenib-resistant HCC [127].

### 3.4 New Strategy for Treating HCC from the Perspective of Cholesterol Metabolism

The norepinephrine reuptake inhibitor maprotiline could significantly reduce the phosphorylation level of SREBP2 through the ERK signaling pathway, reduce cholesterol biosynthesis, and thus inhibit tumor generation [128]. Beyond modulating sorafenib efficacy, cholesterol is functionally repurposed as a drug delivery vehicle. Recent studies demonstrate that polyethylene-cholesterol conjugates self-assemble into polymeric nanocarriers capable of encapsulating sorafenib and other hydrophobic agents [129]. Cholesterol dysregulation further contributes to lenvatinib resistance by remodeling cell surface lipid raft topology, which modulates ATP-binding cassette subfamily B member 1 (ABCB1) activity. This enhanced efflux machinery potentiates drug resistance through accelerated exocytosis [130]. Caspase-3 could regulate the cleavage of SREBP2, promote cholesterol synthesis, and activate the Sonic Hedgehog signaling pathway, thereby increasing the resistance of liver cancer to Lenvatinib [131]. Statins exert anti-HCC effects primarily through cholesterol pathway modulation. By inhibiting HMG-CoA reductase, they suppress mevalonate pathway flux—reducing cholesterol and dolichol biosynthesis—which dysregulates cellular processes (growth, differentiation, apoptosis) to constrain oncogenic progression [132]. Statins may block the lifecycle of HBV and HCV by inhibiting cholesterol synthesis and virus replication, which could potentially prevent their transmission and further liver damage [133]. Genkwadaphnin (GD), a diterpenoid from *Daphne genkwa* (Thymelaeaceae), suppresses hepatocellular carcinoma progression by inhibiting DHCR24. This enzyme blockade disrupts cholesterol biosynthesis and lipid raft integrity, ultimately impeding HCC cell growth and invasion [134]. Concomitant application of lovastatin—a cholesterol biosynthesis inhibitor—to DHCR24-overexpressing HCC cells confirmed cholesterol’s pivotal role in driving oncogenic growth and invasion. These findings substantiate cholesterol reduction as a viable therapeutic strategy for HCC intervention.

Targeting lipid metabolism in HCC cells is a promising anti-cancer strategy. Many new pathways and drugs have been developed to treat HCC, and clinical research is currently underway (Table 1)

**Table 1:** New drugs of HCC treatment with lipid metabolism

Lipid metabolism types	Representative drugs	Possible mechanisms
	Brusatol	Inhibiting NRF2 and enhancing the therapeutic effect of sorafenib by improving lipid metabolism disorders and promoting redox homeostasis [121].
	Etomoxir	Enhancing the therapeutic effect of sorafenib by inhibiting mitochondrial fatty acid oxidation [135].
Fatty acid metabolism	Betulin	Reducing adverse reactions of sorafenib and improving efficacy by blocking SREBP1 [136].
	Orlistat	Inhibiting FASN to improve sorafenib resistance [120].
	Fenofibrate	Activating PPAR in other tumors α to improve the efficacy of cancer vaccines [137].
	D609	Inhibiting SMS1 and enhancing the efficacy of sorafenib by reducing RAS activity [125].
	Batuximab	Inhibiting tumor growth by blocking tumor angiogenesis and activating anti-tumor immunity [138].
Sphingolipid metabolism	Caspase-3	Enhancing the resistance of HCC to lenvatinib by promoting cholesterol synthesis [131].
	Statin	Inhibiting cholesterol synthesis and HBV and HCV replication, blocking the lifecycle of the virus S [139].
	Genkwadaphnin	Inhibiting DHCR24-mediated cholesterol biosynthesis and lipid raft formation, inhibiting the growth and invasion of HCC cells [132].
Cholesterol metabolism	Lycorine	Enhancing the therapeutic effect of sorafenib by inhibiting SCAP to reduce intracellular cholesterol levels [140,141].
	Simvastatin	Effective inhibition of HCC proliferation in combination with PD-L1 antibody [142].

Note: NRF2, nuclear factor erythroid 2-related factor 2; SREBP1, sterol regulatory element-binding protein 1; PPAR, peroxisome proliferator-activated receptor; SMS1, sphingomyelin synthase 1; HCC, hepatocellular carcinoma; HBV, hepatitis B virus; HCV, hepatitis C virus; DHCR24, 24-dehydrocholesterol reductase; SCAP, SREBP cleavage-activating protein.

## 4 Prospects

Lipid metabolism critically sustains HCC progression by supplying bioenergetic and biosynthetic substrates for neoplastic proliferation, invasion, and metastatic dissemination. As the central hub of lipid homeostasis, the liver manifests HCC-specific lipidomic signatures that unveil pathogenic mechanisms. Delineating such metabolic reprogramming uncovers actionable therapeutic targets and informs clinical strategies. Notwithstanding therapeutic advances, persistent obstacles include dose-limiting toxicities requiring treatment de-escalation, alongside acquired resistance rooted in tumor heterogeneity and clonal evolution. Confronting these challenges necessitates developing low-toxicity, high-efficacy modalities targeting metabolic vulnerabilities.

## Data Availability

Data sharing not applicable to this article as no datasets were generated or analyzed during the current study.

## Abbreviations

Abbreviation: Full Term
ACSL4: acyl-CoA synthetase long chain family member 4
MUFA: monounsaturated fatty acid
ACC: acetyl-CoA carboxylase
ACLY: ATP citrate lyase
CPT1: Carnitine Palmitoyl Transferase 1
FASN: fatty acid synthase
MCAD: Medium chain acyl-CoA dehydrogenase
LCAD: long chain acyl-CoA dehydrogenase
CREBP: cAMP-response element binding protein
LPL: Lipoprotein Lipase
ACSM5: acyl-CoA synthetase medium-chain family member 5
SPT: serine palmitoyl transferase
KSR: 3-Ketosphinganine Reductase
CerS: ceramide synthase
DES: dihydroceramide desaturase
SMPD: Sphingomyelin Phosphodiesterase
SMS: Sphingomyelin synthase
SK: Sphingosine kinase
SIPP: Sphingosine phosphatase
